# In search of evidence for the experience of pain in honeybees: A self-administration study

**DOI:** 10.1038/srep45825

**Published:** 2017-04-04

**Authors:** Julia Groening, Dustin Venini, Mandyam V. Srinivasan

**Affiliations:** 1 The Queensland Brain Institute, University of Queensland, Brisbane QLD 4072, Australia; 2 School of Psychology, University of Queensland, Brisbane QLD 4072, Australia; 3 School of Information Technology and Electrical Engineering (ITEE), University of Queensland, Brisbane QLD 4072, Australia; 4 ARC Centre of Excellence in Vision Science, University of Queensland, Brisbane QLD 4072, Australia

## Abstract

Despite their common use as model organisms in scientific experiments, pain and suffering in insects remains controversial and poorly understood. Here we explore potential pain experience in honeybees (*Apis mellifera*) by testing the self-administration of an analgesic drug. Foragers were subjected to two different types of injuries: (i) a clip that applied continuous pressure to one leg and (ii) amputation of one tarsus. The bees were given a choice between two feeders, one offering pure sucrose solution, the other sucrose solution plus morphine. We found that sustained pinching had no effect on the amount of morphine consumed, and hence is unlikely to be experienced as painful. The amputated bees did not shift their relative preference towards the analgesic either, but consumed more morphine and more solution in total compared to intact controls. While our data do not provide evidence for the self-administration of morphine in response to pain, they suggest that injured bees increase their overall food intake, presumably to meet the increased energy requirements for an immune response caused by wounding. We conclude that further experiments are required to gain insights into potential pain-like states in honeybees and other insects.

Which animal taxa have evolved the capacity to experience pain is an ongoing debate. Pain is defined as ‘an aversive sensation or feeling associated with actual or potential tissue damage’^1^. All animal groups seem capable of nociception^2^, but it is often not clear to what extent this reflex response to a noxious stimulus is accompanied by a feeling of pain, discomfort or suffering^3^. While there is increasing evidence that pain occurs in all vertebrates^3^,^4^,^5^, pain and suffering in invertebrates remains a poorly researched and controversial topic^6^,^7^.

Fruit flies, nematodes and honeybees are frequently used as scientific models and subjected to potentially harmful *in*-*vivo* procedures, such as invasive electrophysiology or manipulation of body parts (e.g. refs 8 and 9), but their potential for experiencing pain is rarely considered. Due to the common notion that invertebrates have a limited capacity to suffer^4^, they are usually not included in animal welfare legislation and ethics guidelines (e.g. refs 10 and 11). Only cephalopods have recently been added to the list^12^ due to their complex behaviour and learning ability, which is comparable with lower vertebrates. However, highly plastic behaviour and complex learning and memory have been documented in several other invertebrate taxa as well, such as bees, flies, spiders and slugs^4^,^13^, and evidence from behavioural studies suggests that some crustaceans and molluscs feel pain^14^. Furthermore, invertebrates and vertebrates have much more in common in terms of neurochemistry and physiological responses to injury than previously believed^4^,^15^. Yet it is clear that more data on negative emotional states and pain experience in invertebrates is needed^3^,^6^,^16^. Our study addresses this important but neglected topic and aims to provide a first step towards exploring the possibility of the experience of pain in an insect, the European honeybee (*Apis mellifera*). The honeybee is a promising model organism for the investigation of potential pain due to its impressive learning abilities and cognitive capacities which are in many respects comparable to those of vertebrates^17^,^18^. Honeybee foragers can learn complex associations between odours and appetitive as well as aversive stimuli^19^, will stop other bees from dancing if they are advertising a dangerous food source^20^, and can even exhibit negative emotional states^21^,^22^. Moreover, self-medication^23^, as well as altruistic self-removal of sick foragers^24^ have been reported, suggesting a certain level of self-awareness, if not consciousness, at least in its most basic form^25^.

It is difficult to demonstrate that an animal experiences pain and not just nociception. In fact, some authors argue that it is impossible to prove (or disprove) pain experience in any species, and we may only be able to draw conclusions about the relative likelihood that an animal experiences pain-like states^3^,^7^. Pain is a negative emotional state or feeling^26^ that cannot be measured directly^4^,^27^. Animals cannot tell us what they feel, hence we rely largely on behavioural responses, often combined with physiological and cognitive measures, to infer emotional states and assess the capacity to suffer in other species. Several authors have proposed a list of criteria that need to be fulfilled to demonstrate pain experience in animals^3^,^16^,^27^. One of them is the ‘responsiveness to opioids, analgesics and local anesthetics’^16^. In mammals, the release of endogenous opioids reduces the occurrence of indicators of pain, and so does the injection of the opiate morphine. This effect can be reversed by naloxone, an opiate antagonist^16^. Thus, the presence of opiate receptors and response to analgesics in presumably painful conditions can be a useful indicator of pain experience in animals^27^. It has been demonstrated in praying mantis^28^, crickets^29^ and honeybees^30^ that morphine injection reduces their defensive response to a noxious stimulus in a dose-dependent way and that this analgesic effect can be blocked by naloxone. These studies suggest that insects have opiate binding sites or opioid general sensitivity similar to vertebrates^4^,^31^ or, alternatively, non-opioid receptors to which morphine binds^32^. In honeybees, morphine suppresses the stinging response to electric shock^30^, and it has been suggested that their alarm pheromone (isopentyl acetate) produces stress-induced analgesia^33^.

In addition to analyses of neurochemical and physiological responses to putatively painful stimuli, behavioural studies provide important insights into the potential experience of pain in animals^16^. A particularly convincing experimental approach is the self-administration of an analgesic drug^26^. Self-selection models are well-established. For instance, Colpaert *et al*.^34^,^35^ used a simple choice procedure and found that rats suffering from arthritis would self-administer an anti-inflammatory analgesic (suprofen) or the opiate fentanyl, while sound rats preferred to drink sugar solution. This finding is consistent with the hypothesis that arthritis is painful for rats. Similarly, Danbury *et al*.^36^ showed that lame broiler chickens selectively chose food that contained carprofen as an analgesic, suggesting that they were in pain. The wounded birds consumed more drugged food than sound birds, and the consumption of the analgesic increased with the severity of the injury.

We conducted a similar food-choice experiment to explore potential pain experience in European honeybees. By measuring their food consumption, we examined whether foragers choose to self-administer an analgesic drug in their food when they are subjected to putatively painful treatments. Two different types of injuries were tested: (i) a continuous pinch to the hind leg, and (ii) amputation of part of one middle leg.

We hypothesised that if injured bees experience pain and are given a choice between pure sucrose solution and sucrose solution plus morphine, they would show an increased preference for the analgesic, compared to sound bees.

## Results

For each experiment 540 bees were used (30 injured and 30 control bees in each of the nine replicates), but some individuals died and/or lost the clip that was used to pinch their leg (see Methods). Sample sizes (number of bees and cages used in both experiments, mortality rates and lost clips) are detailed in Supplementary Table S1. The results of all statistical tests are given in Supplementary Table S2.

### Experiment 1 (Clip)

**Figure 1:**
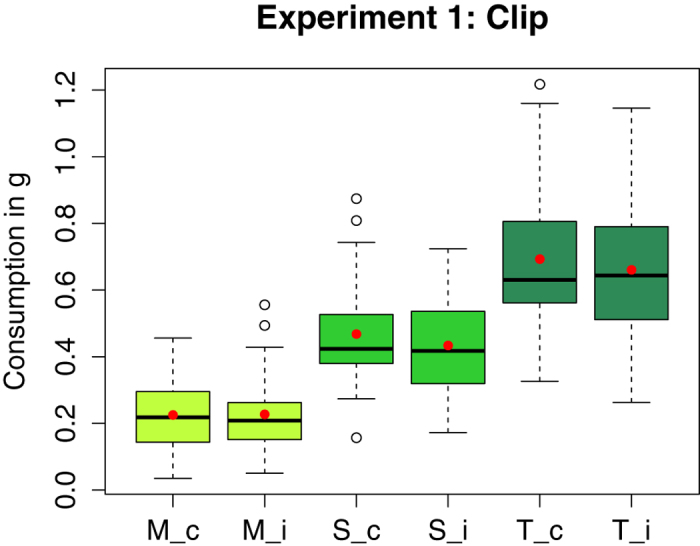
Absolute consumption (in g, per bee, over four days) of morphine solution (M), pure sucrose solution (S) and both solutions combined (total consumption: T) for the control (c) (n = 51) and the injured (i) (n = 49) group of Experiment 1 (n = number of cages). Mean and median values are represented by red dots and horizontal black lines, respectively. No significant difference in consumption of solutions (morphine, sucrose and total) was found between the injured bees and their controls (see Supplementary Table S2).

**Figure 2:**
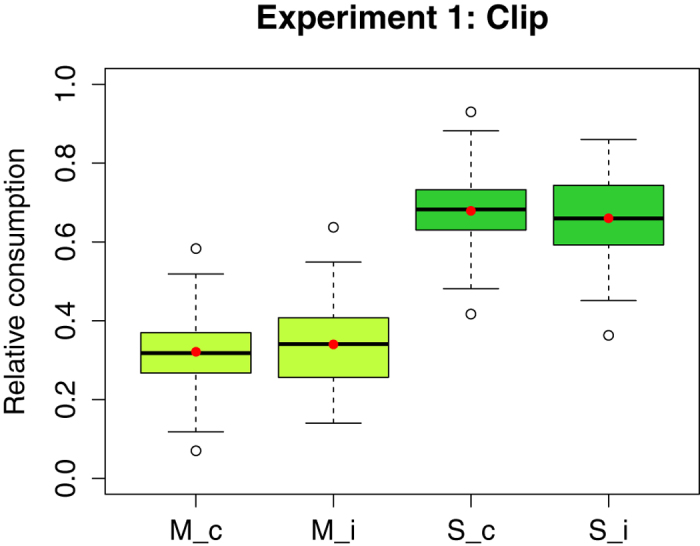
Relative consumption (per bee, over four days) of morphine (M) and pure sucrose solution (S) for the control (c) (n = 51) and the injured (i) (n = 49) group of Experiment 1 (n = number of cages). Mean and median values are represented by red dots and horizontal black lines, respectively. There was no significant difference in the relative consumption of the two solutions between the injured bees and the controls (see Supplementary Table S2).

The absolute consumption of solutions did not differ significantly between the control and the injured bees in Experiment 1 (Fig. 1). The average morphine consumption per bee was identical in both groups, to within the measurement resolution (W = 1265, P = 0.918), and there was also no significant difference in the bees’ sucrose intake (W = 1412, P = 0.264) or in their total consumption (i.e. morphine solution and pure sucrose solution combined, W = 1345, P = 0.512). Correspondingly, the relative consumptions of the two solutions did not differ significantly between control bees and those with leg clips (W = 1177 (morphine), W = 1322 (sucrose), P = 0.620). The total intake of bees from both groups consisted of approximately 30% morphine solution and 70% pure sucrose solution (Fig. 2).

The mortality rate was equally high (18%) for the control and the injured group, and the proportion of cages in which bees died was similar for both treatments (ca. 60%, χ^2^ = 0.0256, P = 0.873; Supplementary Fig. S1). In most of these cages, only one bee died, and the maximum number of dead bees per cage was three (see Supplementary Table S1).

It should be noted that in 26 of the 49 cages that contained injured bees, one to two bees lost their clips, sometimes together with parts of their leg (see Supplementary Table S1). Although all clips were manually attached with great care and by the same person (DV) in order to standardize the procedure as much as possible, we cannot entirely rule out the possibility that some clips may have fallen off because they were too loose. We observed, however, that some individuals were stepping on their clip and pushing it down with another foot, presumably in an attempt to remove it. Occasionally a bee’s leg clip got stuck at the edge of the petri dish underneath the feeder, causing the bee to pull at it and possibly tear off a part of its leg, together with the attached clip. This is in agreement with our observations in previous preliminary experiments, which revealed that bees that were being pinched continuously with clamping tweezers, frequently tore off the pinched leg by intense pulling or circular body motion (unpublished data).

### Experiment 2 (Amputation)

**Figure 3:**
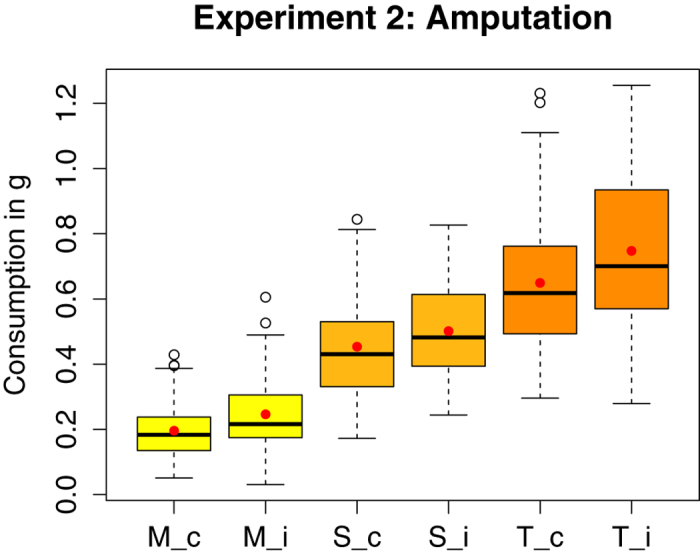
Absolute consumption (in g, per bee, over four days) of morphine solution (M), pure sucrose solution (S) and both solutions combined (total consumption: T) for the control (c) (n = 54) and the injured (i) (n = 52) group of Experiment 2 (n = number of cages). Mean and median values are represented by red dots and horizontal black lines, respectively. The amputated bees ingested significantly more morphine than their controls and also drank significantly more solution in total. There was no significant difference in sucrose consumption between the two groups (see Supplementary Table S2).

**Figure 4:**
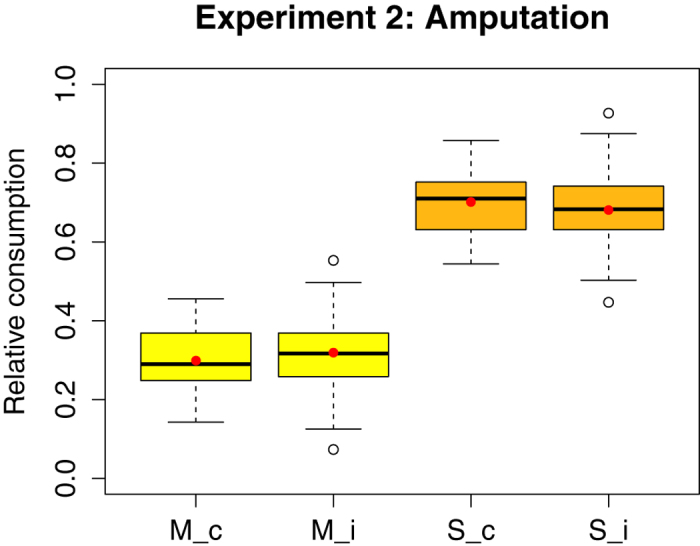
Relative consumption (per bee, over four days) of morphine (M) and pure sucrose solution (S) for the control (c) (n = 54) and the injured (i) (n = 52) group of Experiment 2 (n = number of cages). Mean and median values are represented by red dots and horizontal black lines, respectively. There was no significant difference in the relative consumption of the two solutions between the injured bees and the controls (see Supplementary Table S2).

On average, the amputated bees ingested significantly more morphine solution than the corresponding controls (0.25 g vs 0.20 g, W = 1000, P = 0.011), and also drank more solution in total (0.75 g vs 0.65 g, W = 1051, P = 0.026), but there was no significant difference in the intake of the pure sucrose solution (W = 1145, P = 0.102; Fig. 3). The relative consumption of solutions did not differ significantly between the two groups (W = 1192 (morphine), W = 1616 (sucrose), P = 0.181; Fig. 4). All bees consumed ca. 30% morphine solution and 70% pure sucrose solution.

Mortality was more than twice as high in the injured group compared to the control (12% vs 5%, χ^2^ = 7.6028, P = 0.006; Supplementary Fig. 2). Amputated bees died in almost 50% of their cages, whereas deaths occurred in only 22% of the cages of the control group (χ^2^ = 5.7397, P = 0.017; Supplementary Fig. 2). As in Experiment 1, the highest mortality was three bees per cage, and in the majority of the affected cages four of the five bees survived (see Supplementary Table S1).

## Discussion

Over the last decades there has been growing interest in examining the potential for the experience of pain in insects^7^,^37^, and in investigating the underlying mechanisms. In this study we have used a simple food choice experiment to explore potential pain experience in honeybees. We examined whether foragers alter their food consumption and choose to self-administer an analgesic drug in response to injury, in a way that is consistent with the idea of pain.

We found, firstly, that all of the bees in our study showed a clear preference for pure sucrose solution over morphine. Irrespective of the feeder colour (see Supplementary Information), and across both treatments and experiments, the morphine to sucrose ratio stayed approximately the same (30% vs 70%). Like most insects, honeybees show dietary self-selection behaviour and regulate their food intake according to their nutritional needs^38^,^39^,^40^. It has been shown that caged honeybees make dietary decisions at the individual level and balance their diet by switching between foods to maintain their intake target^40^. Presumably, bees consumed less morphine solution compared to the pure sucrose solution in our experiments, because morphine has a bitter taste^41^ like quinine, which is considered to be distasteful for bees and is commonly used in aversive learning studies^42^. The morphine solution contained slightly less concentrated sucrose than the pure sucrose solution, which might have also affected the bees’ choices.

As no consistent colour preference was found (see Supplementary Information), it appears that the bees in our experiments were able to learn to associate the feeder content with its colour, irrespective what the colour happened to be. While it is possible that the bees used spatial or gustatory cues as well, this demonstrates that they were able to discriminate the two feeders (and their content), which is a necessary prerequisite for this self-administration study. It is known that chronic morphine treatment can diminish associative memory in honeybees, but short-term oral morphine administration does not seem to impair learning and memory^41^.

It is clear that the manipulations tested in Experiment 1 and 2 had different effects on the absolute intake of solutions: While there was no significant difference in the morphine and sucrose consumption between the control and the injured bees in Experiment 1, the amputated bees in Experiment 2 consumed more morphine solution and more solution in total than their controls. As the leg clip in Experiment 1 did not affect the bees’ consumption in any way and also did not increase the mortality compared to their control group, there is no indication that the clip was detrimental or painful for the bees. Although some individuals actively tried to remove this foreign object from their leg, in most bees there was no apparent change in behaviour caused by the clip, which was small and lightweight enough to not restrict their movement. The intention of the leg clip was to create the sensation of a continuous pinch, similar to an attack of a biting predator or competitor^20^,^43^. In preliminary experiments we tested the reaction of (awake) bees to pinching their legs with various clip designs and tweezers, which consistently resulted in a strong defensive response (unpublished data). In the case of the leg clips used in Experiment 1, the mechanical pressure may not have been strong enough to elicit a defensive or aggressive response once the bees woke up from cold anaesthesia. It is also possible that habituation of the relevant sensory pathways during chill coma diminished the sensation of the noxious stimulus when the bees recovered.

In contrast to the bees with leg clips, the amputated bees in Experiment 2 ingested significantly more morphine solution compared to their controls. However, they also showed a slightly elevated sucrose consumption and a higher consumption in total. Hence injured and sound bees ingested the morphine and pure sucrose solution in similar proportions. As the amputated bees did not shift their preference towards morphine, our data do not support the hypothesis that the bees experience this injury as painful and seek relief from it by self-administering the analgesic. Still, the general pattern of increased consumption of liquids in the amputated bees is an interesting finding. It could be interpreted as a compensation for fluid loss after wounding, however no haemolymph leakage was visible at the site of injury after amputation. Instead, the increased intake of these high caloric solutions more likely reflects elevated energy requirements in the amputated bees compared to the corresponding controls. Erler *et al*.^44^ found that wounding elicits the innate immune system of bumblebees and leads to a significant increase of antimicrobial peptide (AMP) expression. Similarly, adult honeybees respond to microbial infections with the synthesis of AMPs. Furthermore, wounding and septic injury induce the activation of prophenoloxidase in honeybees and other insects, which ultimately leads to wound healing and encapsulation of microbes via melanin synthesis^45^. It is well established that the activation of the immune response is costly and requires energy, and it has been shown that food availability can have an impact on the immune defence in insects^38^,^46^. Thus it is likely that in our experiment amputation prompts an immune response, which entails increased energetic demands in these injured bees.

Amputation can be expected to cause more tissue damage and constitute a more severe injury than the leg clip. This appears to be reflected in the amputated bees’ elevated total consumption of liquids as well as their increased mortality, which was almost twice as high compared to their controls. In contrast, in the Clip Experiment, the mortality rate was nearly identical in the injured and the control groups (see above).

It is clear that further studies are needed to explore the possibility of pain experience in honeybees. Future experiments could include the measurement of physiological changes in response to injury, but to distinguish nociception from pain, the greatest insights can be gained by behavioural observations^1^. In particular, avoidance learning or protective motor reactions indicate a more complex response which implies central processing, rather than a simple nociceptive reflex. While bees are capable of avoidance learning^22^,^47^, so far there are no reports of prolonged grooming that is clearly caused by injury and is specifically directed to injured body parts. Another approach would be to investigate trade-offs between stimulus avoidance and other motivational requirements, which reveal central decision making and sufficient cognitive capacities that make pain experience likely^16^. For example, it has been shown that Asian honeybee (*Apis cerana*) foragers evaluate food quality and predation risk^22^, and hence seem to balance two conflicting motivations: the need to collect resources for the colony, and the need to avoid injury or death. As another example, a honeybee that has returned from a food source where it has been attacked by a predator will stop other bees from dancing to advertise that particular food source^43^,^48^. Furthermore, Asian honeybees tune their stop signal production according to the severity of the perceived threat^48^. While these examples do not unequivocally demonstrate the existence of a pain experience, they are intriguing enough to underscore the need for continued investigation of the possibility of pain in invertebrates.

## Methods

Honeybee foragers (*Apis mellifera*) were caught from three different colonies located at the University of Queensland (Brisbane, Australia). Foragers were attracted with tissues soaked in sugar water, placed at the hive entrance. Bees were then captured in tubes and moved into the freezer compartment of a refrigerator (ca. 4–8 min at −10 deg C). Once immobile, they were assigned to two different groups: an injured group and a control group. Bees of the injured group underwent leg manipulations under cold-anaesthesia, while the control bees experienced cold-narcosis only. Two different leg manipulations were tested: attachment of a clip that applied continuous pressure to one hind leg (Experiment 1), and amputation of the middle left tarsus (Experiment 2).

### Experiment 1

In the Clip Experiment, a small aluminium strip (7 × 3 × 1 mm) was crimped around the basitarsus of the bee’s hind leg using pliers, in order to create the sensation of a constant pinch. The clip had an average weight of 0.02 g, light enough for the bees to carry and even fly. It was attached to either the left or the right hind leg, alternating randomly between individuals. The hind leg was chosen as it provides the largest surface to attach the clip, and the bee’s movement is not restricted by the clip in this position. Moreover, the clip was intended to simulate an attack by a competing forager or predator, which often targets the bees’ legs^43^.

### Experiment 2

In the Amputation Experiment, a more severe injury was applied by cutting the left middle leg at the tibiotarsal joint with microdissection spring scissors. The middle leg was chosen under the assumption that a missing leg in this position minimally impairs locomotion and foraging activity.

The control and injured bees were housed separately in small, purpose-built cages made from corflute sheets and mesh (24 × 13.5 × 14.5 cm). The cages contained five bees each (from a given colony, see below) and were set up in randomised order inside the climate controlled (25–26 deg C, humidity 60%) ‘All Weather Bee Flight Facility’ of the Queensland Brain Institute between November 2015 and April 2016. In each cage, two differently coloured feeders (blue and yellow) were provided, one containing 13 ml pure sucrose solution (50%), and the other 12.5 ml sucrose solution (50%) plus 0.5 ml morphine (DBL^®^ morphine sulfate injection BP, 30 mg/ml). Each feeder was made of a 70 ml container (Sarstedt Australia, height 5.5 cm, diameter 4.4 cm) placed upside down on a circular plastic base plate (diameter 7.5 cm, thickness 0.5 cm) with 24 radial grooves from which the bees could drink the solution. A petri dish was placed under each feeder to capture potential spills or overflowing solutions. In our study, bees are required to match potential pain relief with the content of a particular feeder. To facilitate feeder discrimination, we used two different colours, as bees are known for their ability to distinguish colours and associate them with different food^42^.

Feeder colours and contents alternated between replicates (i.e. in some cages the yellow feeder contained morphine, in others the blue feeder) to control for possible colour preferences of the bees. The choice of morphine concentration was based on the study of Nunez *et al*.^31^, which looked at the dose-dependent reduction of the bees’ stinging response to electric shocks after morphine injection.

For each experiment, nine replicates were conducted. An equal number of individuals from each of the three colonies was used, and bees from different hives were kept in separate cages. Each replicate ran over four days and comprised:

- Six cages with injured bees (clip or amputation, two cages per colony)
- Six cages with control bees (no leg manipulation, two cages per colony)
- Two evaporation controls (feeders only, no bees)

In total 540 bees were used for each experiment (30 injured and 30 control bees per replicate). Not all bees survived for four days, hence we recorded the number of dead bees at the start and end of each day. Likewise, the number of lost clips was noted (see Supplementary Table S1).

The feeder weight was recorded at the start and again at the end of each replicate to determine how much of each solution the bees had consumed in each cage, taking into account the evaporation rate (as indicated by the evaporation control feeders) and the number of bees that survived. To account for the variable bee numbers in the cages, we calculated the morphine and the sucrose consumption per bee for each cage, based on the average number of bees that survived each day. At the end of each replicate, bees were sacrificed by freezing, and all cages and feeders were washed, dried and wiped with 70% ethanol to remove scent marks before reusing them for the next replicate.

### Statistical analysis

of the data was performed using the software RStudio (version 0.98.1062). The (non-parametric) Wilcoxon rank sum test (two-sided) was used to compare the means of the morphine, pure sucrose and total (i.e. morphine plus pure sucrose solution) consumption of the injured bees with the respective values for the control group (‘Control vs Injured’ in Supplementary Table S2). The same test was used to examine if the amount of morphine and sucrose consumed varied with the feeder colour (‘Blue vs Yellow’ in Supplementary Table S2).

The mortality rates of the control and the injured treatments were analysed using Chi-squared tests, looking at (i) the proportion of dead bees, and (ii) the proportion of cages in which bees died.

Cages were excluded from the data-set if

- The value for the calculated consumption was negative (or very close to zero), i.e. the average evaporation value exceeded the actual amount of solution lost in a particular feeder (Experiment 1, control group, n = 3)
- More than two individuals in a cage lost their clip (Experiment 1, injured group, n = 5)
- Bees escaped (Experiment 2, injured group, n = 1)
- A weighing error seemed to have occurred (Experiment 2, injured group, n = 1)

The resulting ‘n’ values (number of individuals and number of cages) are given in Supplementary Table S1.

## Additional Information

**How to cite this article**: Groening, J. *et al*. In search of evidence for the experience of pain in honeybees: A self-administration study. *Sci. Rep.* **7**, 45825; doi: 10.1038/srep45825 (2017).

**Publisher's note:** Springer Nature remains neutral with regard to jurisdictional claims in published maps and institutional affiliations.

## Supplementary Material

Supplementary Information
