# Supplementary material for: In search of evidence for the experience of pain in honeybees: A self-administration study
Source: Sci Rep. 2017 Apr 4;7:45825. doi: 10.1038/srep45825 (PMC5379194; doi:10.1038/srep45825)
Supplement: Supplementary Information [file srep45825-s1.pdf]

In search of evidence for the experience of pain in honeybees: A self-administration study

Julia Groening, Dustin Venini, and Mandyam Srinivasan

Supplementary Information

Table S1. This table gives an overview of the number of bees and cages used in both experiments, including mortality and lost leg clips.

	Experiment 1: Clip		Experiment 2: Amputation	
	**Control**	**Injured**	**Control**	**Injured**
Total number of bees	255	245	270	260
Number of bees alive	208	200	256	228
Number of dead bees	47	45	14	32
Total number of cages	51	49	54	52
Number of cages in which all bees survived	19	20	42	28
Number of cages in which bees died	32	29	12	24
Cages with 1 dead bee	20	15	10	17
Cages with 2 dead bees	9	12	2	6
Cages with 3 dead bees	3	2	0	1
Total number of lost clips	NA	33	NA	NA
Cages with 0 lost clips	NA	23	NA	NA
Cages with 1 lost clip	NA	19	NA	NA
Cages with 2 lost clips	NA	7	NA	NA

Table S2. This table lists the results of all tests performed. Significant results are marked in bold and highlighted in light grey. Wilcoxon = Wilcoxon rank sum test, M = morphine solution, S = pure sucrose solution, T = total solution (M+S).

Experiment / Group	Variable	Test	W / X	df	p-value	mean X	mean Y
**Control vs Injured**							
Clip	M consumption (g)	Wilcoxon	1265	NA	0.918	0.225	0.227
Clip	S consumption (g)	Wilcoxon	1412	NA	0.264	0.468	0.434
Clip	T consumption (g)	Wilcoxon	1345	NA	0.512	0.693	0.661
Clip	% M consumption	Wilcoxon	1177	NA	0.620	0.321	0.340
Clip	% S consumption	Wilcoxon	1322	NA	0.620	0.679	0.660
Clip	% Dead bees	Chi-squared	0	1	1	0.184	0.184
Clip	% Cages w dead bees	Chi-squared	0.0256	1	0.873	0.628	0.592
**Amputation**	**M consumption (g)**	Wilcoxon	1000	NA	**0.011**	0.196	0.246
Amputation	S consumption (g)	Wilcoxon	1145	NA	0.102	0.454	0.501
**Amputation**	**T consumption (g)**	Wilcoxon	1051	NA	**0.026**	0.649	0.748
Amputation	% M consumption	Wilcoxon	1192	NA	0.181	0.299	0.319
Amputation	% S consumption	Wilcoxon	1616	NA	0.181	0.701	0.681
**Amputation**	**% Dead bees**	Chi-squared	7.6028	1	**0.006**	0.052	0.123
**Amputation**	**% Cages w dead bees**	Chi-squared	5.7397	1	**0.017**	0.222	0.462
**Blue vs Yellow**							
Clip: Control	M consumption (g)	Wilcoxon	355	NA	0.581	0.235	0.216
Clip: Control	S consumption (g)	Wilcoxon	237	NA	0.100	0.439	0.496
Clip: Injured	M consumption (g)	Wilcoxon	318	NA	0.713	0.237	0.216
**Clip: Injured**	**S consumption (g)**	Wilcoxon	193	NA	**0.034**	0.387	0.487
Amputation: Control	M consumption (g)	Wilcoxon	399	NA	0.559	0.199	0.192
**Amputation: Control**	**S consumption (g)**	Wilcoxon	170	NA	**0.001**	0.381	0.527
**Amputation: Injured**	**M consumption (g)**	Wilcoxon	492	NA	**0.004**	0.298	0.198
Amputation: Injured	S consumption (g)	Wilcoxon	318	NA	0.730	0.489	0.513

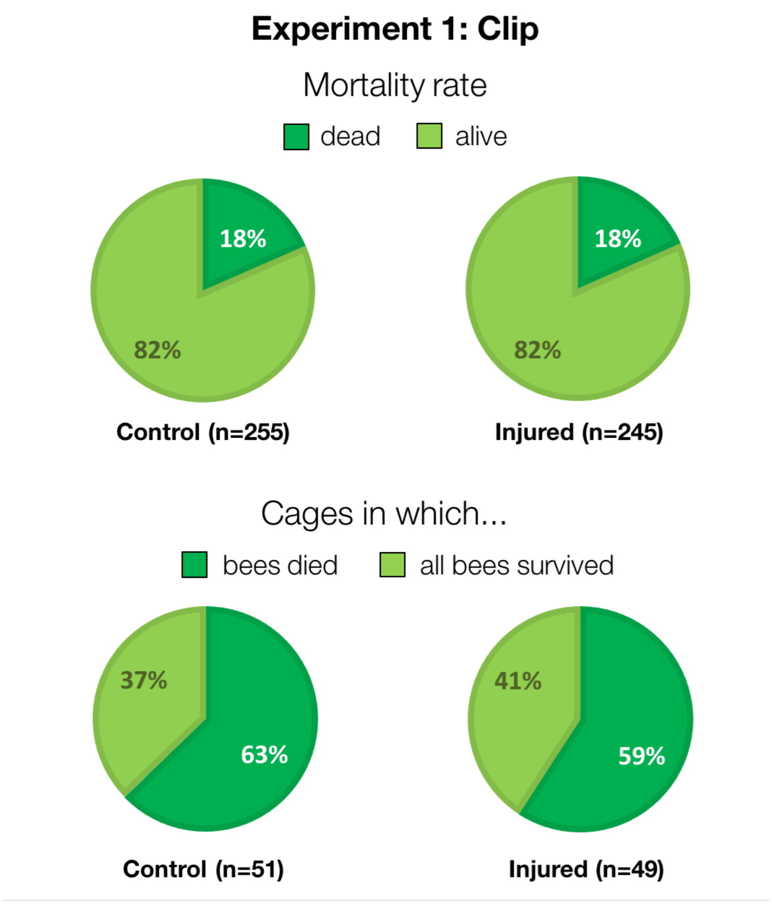

Figure S1. Pie charts comparing the mortality rate of the control and the injured bees (top row; n=number of bees), and the percentage of cages in which bees died (bottom row; n=number of cages) in Experiment 1. Mortality was equally high in the injured and control groups (see Supplementary Table S2).

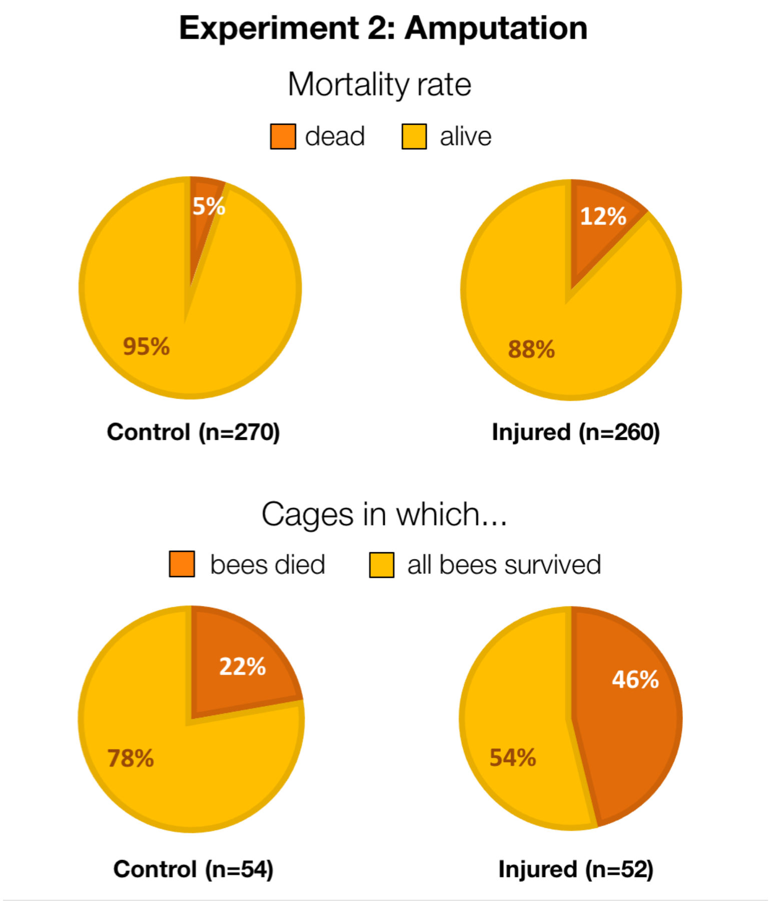

Figure S2. Pie charts comparing the mortality rate of the control and the injured bees (top row; n=number of bees), and the percentage of cages in which bees died (bottom row; n=number of cages) in Experiment 2. Deaths occurred significantly more often in the amputated group than in the corresponding control (see Supplementary Table S2).

Consumption and Feeder Colour

To examine whether the bees' solution intake was influenced by the colour of the feeder, we tested if the amount of morphine and sucrose consumed varied between the blue and the yellow feeders that were used in the experiments.

For the control group of Experiment 1 (Clip), no significant difference in consumption was found between the blue and the yellow feeder (Supplementary Fig. S3). The injured bees, however, drank more sucrose solution when it was offered in the yellow feeder (blue: 0.39g, yellow: 0.49g, W=193, P=0.034; Supplementary Fig. S4).

In the Amputation Experiment, the control bees showed an increased consumption when the yellow feeder contained sucrose solution (blue: 0.38g, yellow: 0.53g, W=170, P=0.001; Supplementary Fig. S5). The injured bees in this experiment displayed no colour-dependent sucrose consumption, but they drank more morphine solution when it was provided in a blue feeder (blue: 0.30g, yellow: 0.20g, W=492, P=0.004; Supplementary Fig. S6).

Taken together, this analysis revealed no consistent colour preference across experiments and groups. Most importantly, the bees showed a preference for sucrose over morphine irrespective of the feeder colour. The morphine to sucrose ratio stayed approximately the same, and this pattern was not overruled when the feeder colours were swapped.

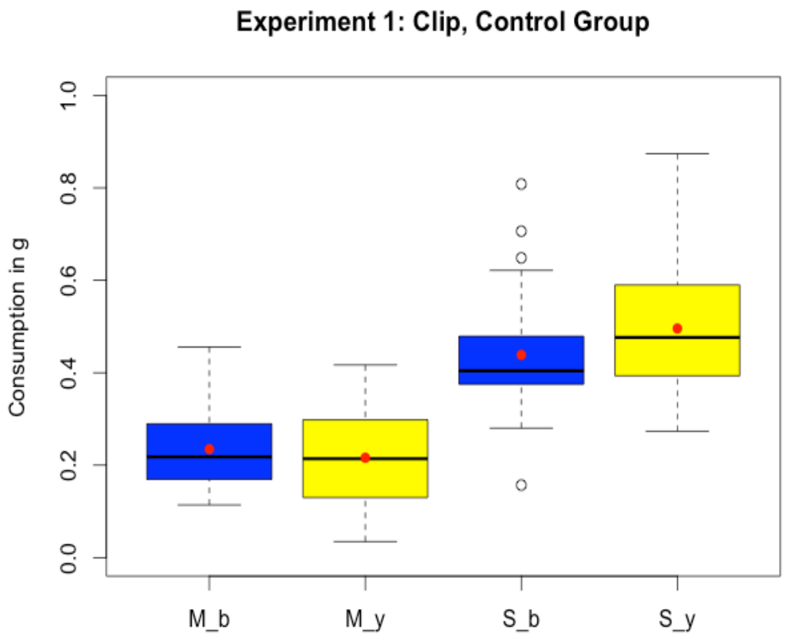

Figure S3. Absolute consumption (in g, per bee, over four days) of morphine solution (M) and pure sucrose solution (S) of the control group of Experiment 1 (Clip) in relation to the feeder colour. Consumption is shown separately for the blue (b) (n=25) and the yellow (y) (n=26) feeders. Mean and median values are represented by red dots and horizontal black lines, respectively. No significant difference in consumption was found between the blue and the yellow feeders, either for the morphine solution or the sucrose solution (see Supplementary Table S2).

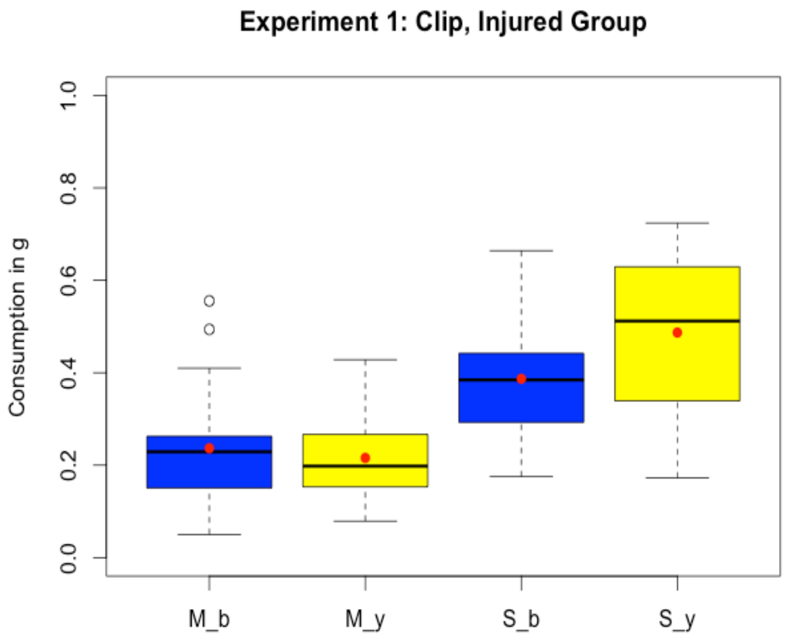

Figure S4. Absolute consumption (in g, per bee, over four days) of morphine solution (M) and pure sucrose solution (S) of the injured group of Experiment 1 (Clip) in relation to the feeder colour. Consumption is shown separately for the blue (b) (n=26) and the yellow (y) (n=23) feeders. Mean and median values are represented by red dots and horizontal black lines, respectively. Bees consumed significantly more sucrose solution when it was offered in the yellow feeder. The morphine consumption did not differ significantly between the blue and the yellow feeders (see Supplementary Table S2).

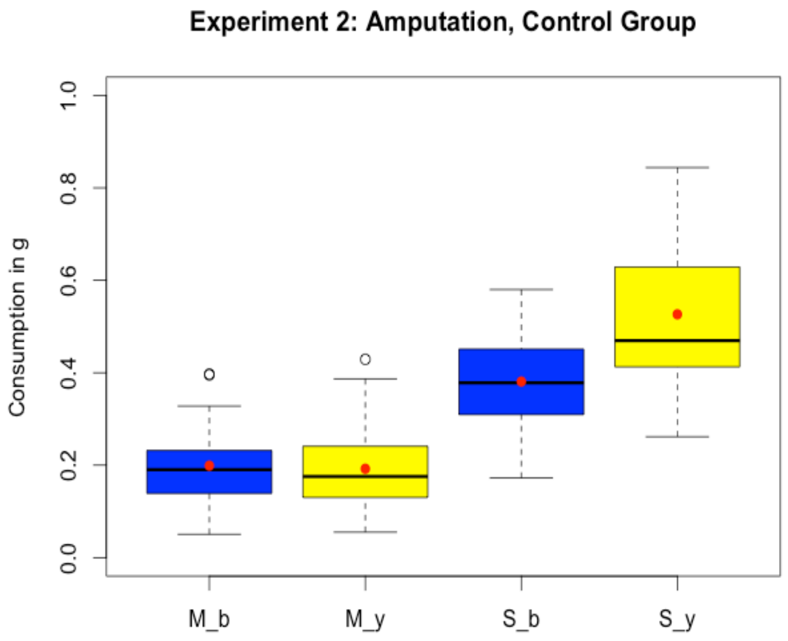

Figure S5. Absolute consumption (in g, per bee, over four days) of morphine solution (M) and pure sucrose solution (S) of the control group of Experiment 2 (Amputation) in relation to the feeder colour. Consumption is shown separately for the blue (b) (n=27) and the yellow (y) (n=27) feeders. Mean and median values are represented by red dots and horizontal black lines, respectively. Bees consumed significantly more sucrose solution when it was offered in the yellow feeder. The morphine consumption did not differ significantly between the blue and the yellow feeders (see Supplementary Table S2).

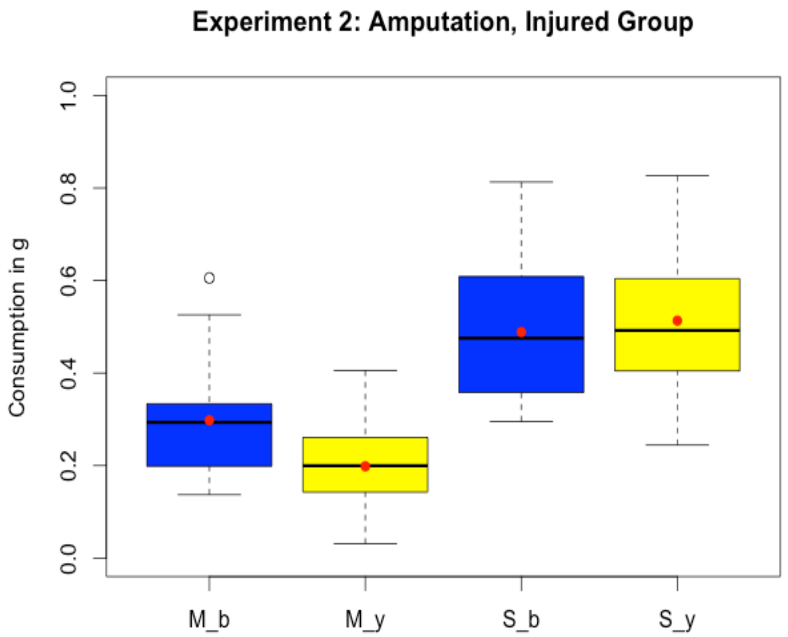

Figure S6. Absolute consumption (in g, per bee, over four days) of morphine solution (M) and pure sucrose solution (S) of the injured group of Experiment 2 (Amputation) in relation to the feeder colour. Consumption is shown separately for the blue (b) (n=25) and the yellow (y) (n=27) feeders. Mean and median values are represented by red dots and horizontal black lines, respectively. Bees consumed significantly more morphine solution when it was offered in the blue feeder. The sucrose consumption did not differ significantly between the blue and the yellow feeders (see Supplementary Table S2).
